# LUMAN/CREB3 Plays a Dual Role in Stress Responses as a Cofactor of the Glucocorticoid Receptor and a Regulator of Secretion

**DOI:** 10.3389/fnmol.2018.00352

**Published:** 2018-09-26

**Authors:** Jenna Penney, Tiegh Taylor, Neil MacLusky, Ray Lu

**Affiliations:** ^1^Department of Molecular and Cellular Biology, College of Biological Science, University of Guelph, Guelph, ON, Canada; ^2^Department of Biomedical Sciences, University of Guelph, Guelph, ON, Canada

**Keywords:** glucocorticoid receptor, LUMAN/CREB3, hypothalamic pituitary adrenal axis, stress, secretion, nuclear receptor co-factor

## Abstract

LUMAN/CREB3, originally identified through its interaction with a cell cycle regulator HCFC1, is a transcription factor involved in the unfolded protein response during endoplasmic reticulum stress. Previously using gene knockout mouse models, we have shown that LUMAN modulates the glucocorticoid (GC) response leading to enhanced glucocorticoid receptor (GR) activity and lower circulating GC levels. Consequently, the stress response is dysregulated, leading to a blunted stress response in the *Luman*-deficient mice. One question that remained was how LUMAN deficiency affected the stress response at the cellular level leading to the changes in the physiological stress response. Here, we found that LUMAN interacts with GR through a putative nuclear receptor box site and can activate GR in the absence of a ligand. Further investigation showed that, when activated, LUMAN binds to the glucocorticoid response element (GRE), increasing the activity of GR exponentially compared to GR-ligand binding alone. On the other hand, we also found that in the absence of LUMAN, cells were more sensitive to cellular stress, exhibiting decreased secretory capacity. Hence our current data suggest that LUMAN may function both as a transcriptional cofactor of GR and a hormone secretion regulator, and through this, plays a role in stress sensitivity and reactivity to stress.

## Introduction

The secretion of glucocorticoids (GCs) is induced by neuroendocrine responses to stress in mammals and has numerous downstream effects. Aberrations in this response have been linked to common mental disorders, such as depression and anxiety, as well as various metabolic diseases and cancers (Pariante, 2009). Understanding the underlying mechanisms of GC dysregulation is key in preventing and treating these diseases. Dissecting and analyzing factors involved in the primary stress response, the hypothalamic pituitary adrenal (HPA) axis, will help gain critical knowledge of these mechanisms. GCs are released following a cascade of events that occur in the HPA axis. Corticotropin releasing hormone (CRH) is released from the hypothalamus, triggering the release of adrenocorticotropin releasing hormone (ACTH) from the anterior pituitary, which acts on the adrenal cortex to initiate the synthesis and secretion of GCs. GCs then negatively regulate their release by inhibiting the secretion of CRH and ACTH from the hypothalamus and pituitary, respectively (O’Connor et al., 2000). One of the primary differences between GCs and CRH/ACTH is that, as steroid hormones, GCs can move freely into cells while CRH and ACTH are peptide hormones which are synthesized, stored in vesicles, and rapidly released when triggered by stress (Stevens and White, 2010).

Secretion takes place at low levels in most cells types, and at extremely high levels in specialized cells, such as cells found in the pancreas, the salivary gland, and various neuroendocrine tissues including the neurosecretory cells in the hypothalamus and pituitary (Barlowe and Miller, 2013). When the stress response is elicited, a number of important changes occur, including increased secretion of CRH, ACTH and GCs, all of which has to occur quickly in order to initiate an appropriate response (Pariante and Lightman, 2008). Importantly, while these hormones are the main effectors of the stress response, many other factors must be selectively upregulated in order to coordinate this response, including enzymes responsible for synthesizing GCs such as 3β-hydroxysteroid dehydrogenase (3βHSD) and 21-hydroxylase (CYP21) (Whirledge and Cidlowski, 2010). Studies in the adrenal gland have shown that behavioral stress causes a rapid upregulation of genes involved in catecholamine synthesis, such as StAR (Prasad et al., 2016). As cells usually operate at the limits of their secretory capacity during times of increased secretory demand, they toned to accommodate the rapid influx of proteins channeled through the ER which triggers adaptive unfolded protein response, in differentiating antibody-secreting B cell for instance (Gass et al., 2002; Hetz and Papa, 2018). Perturbations in the secretion of proteins will also cause stress in the ER and activate the UPR, which may lead to detrimental effects on the cells, including apoptosis (Xu et al., 2005). The mechanism by which secretory capacity is selectively upregulated in specific cell types is not well understood. CREBA is a transcription factor found in *Drosophila* that has been implicated in secretion; the mammalian homologs are proteins belonging to the CREB3 family (Fox et al., 2010). It has been shown that CREBA and CREB3 family members of bZip transcription factors can function to up-regulate expression of protein machinery required in all cell types for basal secretion and in specialized cells with an increased secretory capacity (Dalle et al., 2011). Furthermore, over half of the genes that require CREBA encode identifiable secretory components and phenotypes associated with the loss of CREBA are consistent with the role it plays in secretion (Abrams and Andrew, 2005).

CREB3, also known as LUMAN, is an endoplasmic reticulum (ER) membrane-bound transcription factor that is involved in ER stress and the related UPR (Audas et al., 2008) as well as being involved in the Golgi stress response (Taniguchi and Yoshida, 2017). Cellular stressors can suppress the secretory capacity of cells as well as decrease the efficacy of the protein folding and modification machinery in the ER, leading to an accumulation of misfolded proteins in the ER (Moore and Hollien, 2012). The UPR is a highly conserved mechanism that is activated in response to accumulation of misfolded proteins. The main purpose of the UPR is to restore normal function of the cell by halting protein translation, degrading misfolded proteins, and increasing the production of molecular chaperones involved in protein folding (Schroder and Kaufman, 2005). LUMAN is known to play an important role in cellular stress responses including parts of the UPR. It is also involved in the physiological stress response altering GC and GR activity in mice. LUMAN contains two LxxLL nuclear receptor (NR) binding motifs common in NR co-factors (Luciano and Wilson, 2000; Penney et al., 2017). LUMAN is most highly expressed in the hypothalamus, hippocampus, anterior pituitary and adrenal gland. We have previously shown that *Luman*-deficient mice have low levels of corticosterone and high levels of its receptor, the glucocorticoid receptor (GR), resulting in elevated GR activity (Penney et al., 2017). However, the mechanism by which LUMAN deficiency causes this phenotype is not well understood. The question that remains is whether the initiating event is the increase in GR expression, which would result in the increase in negative feedback in the HPA axis decreasing GC levels; and/or if a secretion defect causes low GCs which could subsequently lead to the compensatory increase in GR expression. It is important to note that these mechanisms are not mutually exclusive.

In this paper, we examine the mechanism behind the role LUMAN plays in regulating GR activity. Here, we present data that indicates LUMAN binds to GR through the LxxLL motif and that it alters GR activity through this interaction, as well as binding to the glucocorticoid response element (GRE). These results indicate that LUMAN acts as both a transcription factor and co-factor leading to alterations in GR activity. We further examined LUMANs role in the cellular stress response and found that in the absence of LUMAN, the cells are more sensitive to cellular stress, leading to decreased secretory capacity of the cell possibly leading to altered GC release. It is clear that LUMAN plays a dual role in the stress response, working at both the whole animal level, as well as the cellular levels.

## Materials and Methods

### Animals

This study followed the Canadian Council of Animal Care guidelines and was approved by the Animal Care Committee at the University of Guelph. The LUMAN gene knockout mouse line was generated in collaboration with the International Gene Trap Consortium (Nord et al., 2006). Chimeric mice were backcrossed to C57BL/6 mice (Charles River, Montreal, QC, Canada) to produce a 99.9% congenic mouse strain. C57BL/6NTac mice were group housed with same-sex siblings and maintained on a 12-h light/dark cycle (10:00–22:00). Temperature was maintained at 21–24°C, and food (2014 Teklad Global 14% protein rodent maintenance diet) and tap water were provided *ad libitum*. To obtain sufficient mice in certain circumstances pups from LUMAN KO/HET, mice were cross fostered onto CD1 dams. Due to low LUMAN KO pup survival, heterozygote mice were used in all experiments except the behavior assays where LUMAN KO mice were used. Mice were euthanized by cervical dislocation and tissues were collected either in liquid nitrogen for protein and mRNA extraction or in 4% Paraformaldehyde for histological analysis.

### Cell Culture

All cell types were grown in monolayer culture in Dulbecco’s modified Eagle’s medium (high glucose) supplemented with 10% (vol/vol) fetal bovine serum (Invitrogen), 100 IU/ml penicillin, and 100 g/ml streptomycin. All cultures were maintained in a 5% CO_2_ humidified atmosphere at 37°C and passaged every 2 to 3 days. Cells were plated 24 h prior to transfection and allowed to grow to 60% confluence prior to transfection. Cells were transfected by polyjet transfection reagent (SignaGen Laboratories) as per the manufacturer’s instruction.

### ELISAs

Blood samples (100 to 150 μl) were collected in the active cycle (1100 to 1300 h) from the saphenous vein of the hind limb; serum was separated and stored at -80°C. Hormone levels were detected using a CRH enzyme-linked immunosorbent assay (ELISA) kit (Aviva Biosystems, San Diego, CA, United States) as per the manufacturers’ instructions, and detected using a POLARstar Omega plate reader (BMG Labtech GmbH, Offenburg, Germany). Statistical analysis was performed using a two-way ANOVA with repeated measures. Data were deemed significant at a *P*-value < 0.05.

### RNA Analysis and qRT-PCR

Total RNA was isolated using Trizol (Invitrogen, Carlsbad, CA, United States) from adult mouse tissues. cDNA was synthesized from total RNA using SuperScriptIII reverse transcriptase (Invitrogen) and oligo(dT) (Roche Diagnostics, Laval, QC, Canada). Transcript levels were measured by quantitative RT-PCR (qRT-PCR) using PerfeCTa SYBR green Supermix with 6-carboxy-X-rhodamine (ROX) (Quanta Biosciences, Inc., Gaithersburg, MD) and primers against the mouse genes. Samples were run on a StepOnePlus Real-Time PCR System (Applied Biosystems, Carlsbad, CA, United States) and subjected to standard curve analysis, and arbitrary values were represented, adjusting for primer efficiencies. For primer sequences see **Supplementary Table S1**.

### Protein Extraction and Western Blot Analysis

Tissues were homogenized in Trizol (Invitrogen, Carlsbad, CA, United States) RNA was extracted and the phenol phase was frozen at -80°C until protein extraction was done. Isopropanol precipitation was performed to isolate the protein. Total protein was quantified using Pierce^®^ BCA protein assay reagent (Thermo Fisher Scientific, United States) according to the manufacturer’s instructions. The blots were visualized using ECL (GE Healthcare, Piscataway, NJ, United States) on Amersham Hyperfilm ECL (GE Healthcare) or using the ChemiDoc XRS + imaging system (Bio-Rad).

### Antibodies

Primary antibodies were used at the following dilutions: GR polyclonal antibody (sc-1004; Santa Cruz) at 1:400, Creb3 polyclonal antibody (Proteintech), Lamin polyclonal antibody (abcam, ab26300) at 1:1000, and Tubulin monocloncal antibody (abcam, ab7291). Secondary horseradish peroxidase (HRP)-conjugated antibodies were used at 1:10,000 (Promega).

### Dual Luciferase Assay

HEK293 cell cultures were grown to approximately 70% confluence prior to transfection using polyjet (SignaGen Laboratories) using manufacturer’s instructions. The cells were co-transfected in a 12-well plate with 0.3 μg of MMTV-luc, 0.3 μg of GR, 0.05 μg of pRL-SV40 (Promega), and 0.2 μg of either pcDNA3.1, pcLUMAN, pc N terminal LUMAN, LxxLL KO N-terminal LUMAN, Δ AD N terminal LUMAN, or ΔDBD N Terminal LUMAN. At 16–18 h post-transfection, the medium was replaced to allow the cells to recover for 8 h. Dexamethasone was then added and incubated for 12 h. The cells were harvested, and dual luciferase assays were carried out according to the manufacturer’s instruction (Promega). Reporter activity was calculated as relative luciferase activity (firefly luciferase/*Renilla* luciferase) to correct for transfection efficiency. Assays were independently repeated at least five times, and results are shown with standard error. Statistical analysis was done using a one-way ANOVA and a Tukey test, the data had to be log transformed to meet the assumptions of normality.

### Cellular Fractionation

HEK293 cells were grown to 70% confluency and transfected with polyehtyleneimine as per manufacturer’s instructions (Santa Cruz, CA), 36 h after transfection the cells with treated with Dexamethasone (100 nm) (or ETOH for control), 12 h after treatment the cells were collected using the Genetex: Fractionation of Membrane/Cytoplasmic and Nuclear Proteins protocol. In brief, cells were collected in cold PBS, spun down, and resuspended in a hypotonic buffer, after a 15-min incubation detergent (NP40) was added, mixed, and the samples were centrifuged, the supernatant was kept as the cytoplasmic fraction. The nuclear pellet was re-suspended in cell extraction buffer, incubated for 30 min after which the sample was centrifuged, and the supernatant was transferred to a new tube as the nuclear fraction. These samples were then either stored at -80 freezer or run immediately on an SDS page gel.

### Coimmunoprecipitation

For lysis and co-immunoprecipitation of various LZIP constructs and the GR, HEK293 cells were transfected with indicated vectors using Polyehtyleneimine as per manufacturer’s instructions (Santa Cruz, CA). Media was changed after 6 h, 40 h after transfection cells were crosslinked using 1% paraformaldehyde (Sigma) for 10 min and were then lysed in RIPA buffer [150 mM NaCl, 1% (V/V) triton x-100, 0.5% (V/V) sodium deoxycholate, 0.1% (V/V) SDS, 50 mM Tris] supplemented with 1 mM PMSF as well as 10 μg/ml aprotinin and leupeptin at 4°C for 10 min. After centrifugation (4°C, 13,000 RPM, 10 min), the indicated antibody was immediately added to supernatant and incubated on a rotator at 4°C for 4 h. Immunoprecipitation was performed using Sera-Mag SpeedBead Protein A/G (GE Healthcare) following the manufacturer’s protocol. The lysates and immunoprecipitates were detected by Western blot using the antibodies indicated by measurement with Pierce ECL Western blotting substrate (Thermo Fischer, Rockford, IL, United States).

### Chromatin Immunoprecipitation

m-Hippo-E14 cells were cultured in 10-cm plates and either left untreated or were treated with 100 nM of DEXamethasonee. The chromatin immunoprecipitation (ChIP) assay was performed using the Chromatrap ChIP-seq Protein G kit following the manufacturer’s instructions. Briefly, after cross-linking in 1% formaldehyde, the cells were lysed and sonicated yielding fragments 200–600 bp. A 10% aliquot of the precleared chromatin was taken as input, and the rest was incubated with either 2 μg of CREB3 (Proteintech) or 2 μg of rabbit IGG followed by immunoprecipitation. After reversing the formaldehyde-induced cross-linking, the chromatin DNA was used in Q-RTPCR, using primers that bind in the promoter region of each gene within 200 bp of a GRE site, for primer sequences see **Supplementary Table S1**.

### Viral Infection and Trafficking

Mouse embryonic fibroblasts were infected with an adenoviral vector expressing YFP–VSV-G^ts0-45^ (VSVG; X. Zha and R. Parks, Ottawa Hospital Research Institute, Ottawa, ON, Canada) and incubated at 40.5°C for 18–20 h to allow VSVG accumulation in the ER. Under treatment cells were incubated with 200 nM Brefeldin A for 3 h. Cells were then transferred to 32°C and incubated for specific time periods (10 min, 20 min, and 1 h) to allow the VSVG to move to the Golgi/PM. The cells were fixed the specific time period using 4% PFA. Cells were subsequently visualized and analyzed with a confocal microscope (Quorum Diskovery Spinning Disk Confocal System). VSVG was considered ER associated if VSVG could be visualized within ER compartments, whereas VSVG was considered Golgi associated once VSVG entirely co-localized with the Golgi marker GM130. Data were expressed as a percentage of total cell number in each condition. Lentiviral infection of Gaussia Luciferase (GLuc) (B. Tannous, Harvard Medical School, Boston, MA) was done in mouse embryonic fibroblasts and the conditioned media was collected and assayed to assess the level of secretion. The efficiency of infection was calculated and used to standardize the data; 48 h after infection, media were changed, and 50-μl media samples were harvested at various time points for analyses for GLuc activity to determine the rate of secretion. For the Gluc assay, statistical analysis was done using a two-way ANOVA *post hoc* Tukey test; for the VSVG a two-way ANOVA *post hoc* Dunnett’s *t*-test was used.

### Immunostaining

Cells were fixed for 5 min in ice-cold methanol and blocked for 60 min in 10% goat serum at room temperature. Antibody incubations (Creb3 1:200, proteintech; Alexa594, anti-mouse IgG 1:400, Molecular Probes and Alexa488, anti-rabbit IGG 1:400) were for 30 min at 37°C with 5% CO_2_. Glass cover slips were mounted in 50% glycerol/500 pmol DAPI solution and sealed with nail polish. Images were visualized with a confocal microscope (Quorum Diskovery Spinning Disk Confocal System).

### Statistical Analysis

All the assays were independently repeated at least three times, and results are shown with standard error. Statistical analysis was done using a one-way or two-way ANOVA with *post hoc* Tukey test or Dunnett’s test, or a two-tailed student *t*-test. Results were considered significant when *p* < 0.05.

## Results

### Luman Alters GR Activity

**FIGURE 1:**
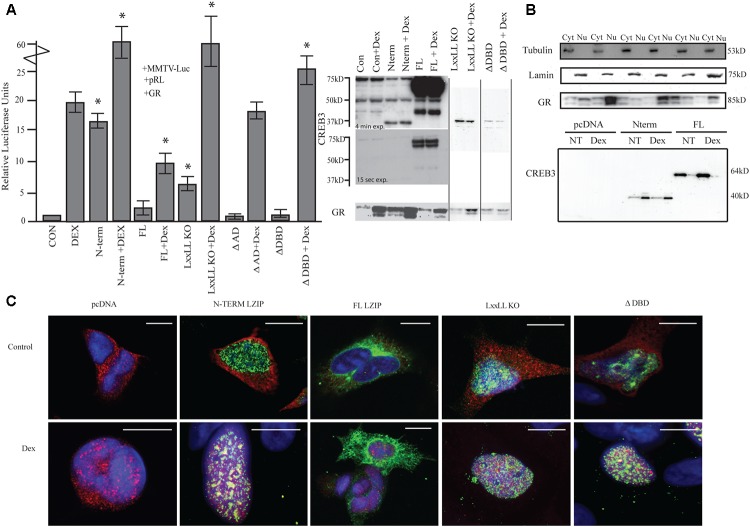
LUMAN alters GR activity. **(A)** Activation of the MMTV promotor by LUMAN. HEK293 cells were transiently transfected with pGL3-MMTV-luciferase reporter together with the reference *Renilla* luciferase plasmid pRL-SV40, GR and indicated effector plasmid. Luciferase values from five independent experiments were normalized to *Renilla* luciferase activity before being referenced to control values. Cell lysates from the luciferase assay were subjected to Western blot analysis using the following antibodies CREB3 (1:1000), GR (G-5) (1:400). Presented as mean ± SEM; ^∗^*p* < 0.05 calculated by a one way and a Tukey test (Untreated compared to pcDNA control, treated compared to pcDNA + DEX). **(B)** A cellular fractionation shows the localization of both GR and LUMAN using Lamin (1:1000) and Tubulin (1:1000) as controls. **(C)** Using immunofluorescence we confirm the localization of GR (red-594) and CREB3 (green-488) as well as looking at the mutant CREB3 constructs (Zoomed out pictures can be seen in **Supplementary Figure S5**). Scale bars: 10 μM.

To assess how LUMAN alters GR activity, a dual luciferase assay was performed in HEK293 cells to allow for the overexpression of various LUMAN constructs. The data shown indicates that N-terminal LUMAN can activate the MMTV (mouse mammary tumor virus) reporter, which contains GREs (Belikov et al., 2001), in the absence of a ligand (DEX) [*t*(4) = 19.313, *p* = 2e^-16^] (**Figure 1A**). When both N-terminal LUMAN and DEX were administered the reporter activity increased dramatically compared to either factor alone [*t*(4) = 5.773, *p* = 1.80e^-05^]. There is no activation of the reporter with full-length LUMAN expression when compared to the control [*t*(4) = 1.390, *p* = 0.175] (pcDNA); however, significant activation was observed once DEX was added [*t*(4) = 1.94, *p* = 0.004] but the effect appeared to be dampened compared to DEX treatment alone [DEX alone vs. DEX + FL: *t*(4) = 4.741, *p* = 0.000163] (**Figure 1A**).

To determine the regions required for activation, various deletion mutants of LUMAN, LxxLL KO (NR box mutant), ΔAD (activation domain), and ΔDBD (DNA binding domain mutated) were tested (**Figure 1A**). The LxxLL KO mutant without treatment was still able to moderately activate the reporter [*t*(4) = 12.161, *p* = 3.99e^-13^]. When treated with DEX, a dramatic increase in activation was seen compared to DEX alone similar to cells overexpressing N-terminal and treated with DEX [*t*(4) = 5.773, *p* = 1.80e^-05^]. With overexpression of ΔAD, no difference in activation was seen in the absence or presence of DEX when compared to the control values (pcDNA and pcDNA + DEX, respectively) [pcDNA: *t*(4) = 0.425, *p* = 0.674; pcDNA + DEX: *t*(4) = 0.703, *p* = 0.491118] (**Figure 1A**). However, the ΔAD mutant appears to produce a very unstable protein that is not detectable through Western blot or immunocytochemical techniques but expression of the construct has been confirmed through q-RT-PCR (**Supplementary Figure S1**). The ΔDBD mutant does not affect GR activity in the absence of DEX [*t*(4) = 1.117, *p* = 0.273], however, under DEX treatment the ΔDBD mutant induces GR activity significantly when compared to DEX alone [*t*(4) = 2.502, *p* = 0.022222]. Interestingly, this enhanced activation seen with the ΔDBD mutant and DEX is significantly lower when compared to N terminal + DEX or the LxxLL KO mutant + DEX [N-term: *t*(4) = 5.48, *p* = 0.002; LxxLL KO: *t*(4) = 7.37, *p* = 0.008] (**Figure 1A**), indicating different mechanisms may be at play.

Through immunostaining (**Figure 1C**) and cellular fractionation (**Figure 1B**), we found that full length LUMAN appeared to be predominantly located in the cytoplasm, while the N-terminal LUMAN, LxxLL KO and ΔDBD were localized in the nucleus. GR was predominantly localized in the cytoplasm until treated with DEX which caused it to translocate to the nucleus (**Figures 1B,C**).

### Luman Binds to GR Through the Putative NR Boxes

**FIGURE 2:**
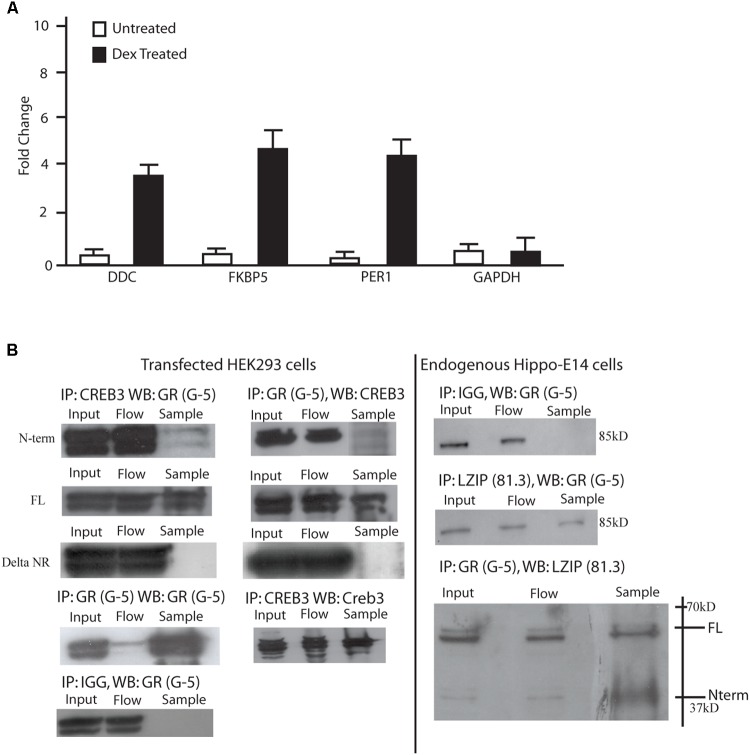
LUMAN binds to GR through the LxxLL motifs. **(A)** Chromatin immunoprecipitations were performed using mHippo-E18 cells showing that with DEX treatment, CREB3 binds to the promoter of FKBP5, Per1, and DDC all containing GREs. Primers for GAPDH and IGG pull downs were both used as negative controls. All data is representative of three independent replicates, presented as mean ± SEM. **(B)** Coimmunoprecipitation experiments were performed in both mHippo-E18 cells **(B)**, looking for endogenous interaction, as well as HEK293 cells transfected with various constructs to help map the interaction. Here we show that GR interacts with both full-length and N-terminal LUMAN but not the LxxLL KO N-terminal LUMAN mutant. CREB3 antibody (proteintech) and GR (G-5 Santacruz) was used to pulldown their respective proteins, IGG was used as a negative control. Full blots can be seen in **Supplementary Figure S6**.

Mouse embryonic day 18 hippocampal cells (m-hippo-E18) were used to search for LUMAN targets due to the relatively high LUMAN expression found in these cells. As well, these cells are physiologically relevant when investigating HPA axis regulation. Using chromatin immunoprecipitation, we found that in un-treated (NT) cells, LUMAN did not bind to any of the GR responsive gene promoters examined. In cells treated with DEX (100 nM), however, LUMAN bound to the promoter region of each of the genes that contain GC respond elements (GREs): period homolog 1 (Per1), and dopamine decarboxylase (DDC) and FK506 Binding Protein 5 (FKBP5) (**Figure 2A**).

To investigate if the LUMAN protein interacts with GR, coimmunoprecipitation experiments were performed to examine endogenous protein interaction in m-hippo-E18 cells (**Figure 2B**), and transfected LUMAN protein interaction with transfected GR protein in HEK293 cells (**Figure 2C**). The results show that LUMAN interacts with both endogenous and transfect GR protein through the NR box.

### Luman-Deficient Cells Have Reduced Secretory Capacity Under Stress

**FIGURE 3:**
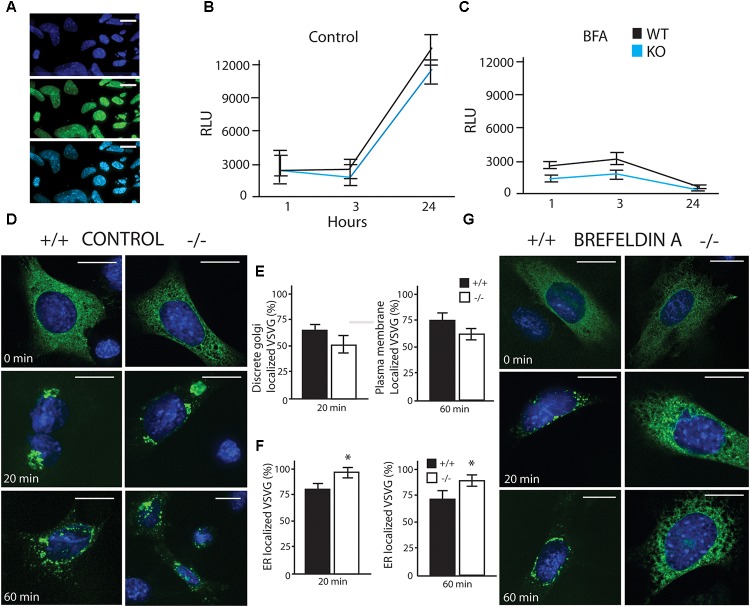
LUMAN –/– cells have reduced secretory capacity under stress. **(A)** Infection efficiency of Gaussia Luciferase (GLuc) virus shown through co-staining with DAPI. **(A–C)** GLuc was delivered to cells using an adenovirus, and its secretion was monitored by measuring luciferase activity in the media at specific time points under control conditions **(B)** as well as treatment with brefeldin A (BFA) **(C)**. GLuc data shown is presented as the average of five independent experiments, showing mean ± SEM; ^∗^*p*<0.05 calculated by a two-way ANOVA, *post hoc* Tukey (*n* = 4). **(D,E)** Trafficking of VSVG was unaffected by removing LUMAN under control or vehicle conditions, **(E)** No difference was seen in Golgi localization after 20 min or plasma membrane localization after 60 min. **(F,G)** An exaggerated delay in trafficking of VSVG from the ER to the Golgi in the absence of LUMAN was shown under 3 h treatment with BFA (200 nM). **(F)** The percent of VSVG stuck in the ER was higher in the KO cells when compared to WT. Representative images shown from three independent experiments completed in duplicate, presented as mean ± SEM; ^∗^*p* < 0.05 calculated by a two-way ANOVA *post hoc* Dunnett’s *t*-test. Scale bar: 10 μM. GM130 Co-staining can be seen in **Supplementary Figure S7**.

To investigate potential differences in cellular secretion, two lines of mouse embryonic fibroblasts (MEFs) were used in these experiments, a wildtype (WT) strain and a *Luman* knockout (KO) strain. We found that LUMAN KO cells were more sensitive to Brefeldin A (BFA), but showed no significant to other stressors (tunacamycin, thapsigargin, monensin, MG132, BFA and DEX– data not shown). In the Gaussia Luciferase (Gluc) assay (**Figures 3A–C**) assay, several ER and Golgi stressors were used (**Figure 3C**). These results indicate that in control experiments (no treatment or mock treatment with ethanol), there was no difference in flux through the secretory pathway [*f*(2) = 0.0307, *p* = 0.8622] (**Figure 3B**). When these cells were treated with a low level of BFA (200 nm), secretion was delayed in the KO cells when compared to WT [*f*(2) = 13.2471, *p* = 0.001188]. (**Figures 3C,G**). These results were consistent with the VSVG assay [Control *f*(2) = 0.5832, *p* = 0.4540] [BFA treated *f*(2) = 20.920, *p* = 0.0001842] (**Figures 3D–G**), in this assay a temperature sensitive VSVG mutant was transduced into the cells which were cultured at 40°C causing the protein to be retained in the ER. Once placed at a permissive temperature (32°C), the protein moved through the secretory pathway and we observed the protein location at different time points. Confirmation that treatment with 200 nM BFA induced LUMAN cleavage is shown in **Supplementary Figure S2** to indicate that LUMAN is active under these conditions.

### Luman Deficiency Impacts the Expression of COPII Vesicle in Response to Stress

**FIGURE 4:**
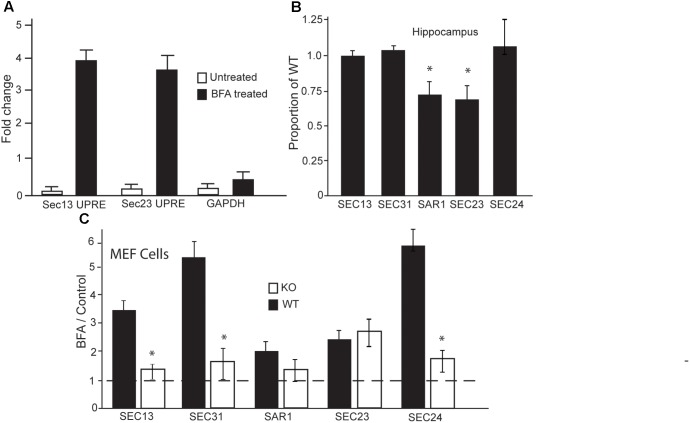
In the absence of LUMAN, the expression of COPII components are altered during stress. **(A)** Chromatin immunoprecipitation was performed using mHippo-E18 cells showing that, when treated with Brefeldin A, LUMAN binds to the promoter region of SEC13 and SEC23 genes. Primers for GAPDH and IGG pull downs were both used as negative controls. **(B,C)** Transcript levels of the COPII components were measured from RNA extracted from the hippocampus **(B)** of wildtype and Luman-deficient mice, data represented as a mean proportion of WT ± SEM. The expression level of the same transcripts were measured in RNA collected from MEF cells. **(C)** Transcript level is presented as a ratio of BFA treated/control; where the controls for WT and KO samples were untreated/vehicle samples from WT and KO cells, respectively, to show the effect of BFA on these components. This data was collected from extracted RNA reverse transcribed into cDNA and analyzed using Q RTPCR. The dotted line indicates all control data were set to one. ^∗^*p* > 0.05 calculated using a Two-way ANOVA *post hoc* Tukey (*n* = 4).

Given that BFA treatment is the only condition under which we observed differences in secretion, and BFA is known to inhibit the formation of COPII vesicles, we wanted to investigate if LUMAN is involved in the formation of these vesicles. To investigate if LUMAN affects COPII vesicle formation we assessed the expression of the major components that make up these vesicles in relation to LUMAN levels. Chromatin immunoprecipitation was performed using m-Hippo-E18 cells under control conditions (no treatment or ethanol) and cells treated with BFA (1 μM) (**Figure 4A**). Under control conditions, LUMAN was not bound to the promoter region of any genes that were examined, however, under treatment with BFA, LUMAN bound to the UPRE-containing gene promoters. Each gene examined (shown in **Figure 4**) encodes a protein that is an integral component of COPII vesicles. The expression of the five main components of COPII vesicles were then examined using qRT-PCR from both the hippocampi of WT and *Luman*-deficient mice (**Figure 4B**), as well as WT and KO MEF cell lines (**Figure 4C**). Without treatment, only Sec23 and SAR1 genes showed a significant difference between *Luman*-deficient and WT samples of the mouse hippocampus (**Figure 4B**) [SAR1: *t*(3) = 4.64, *p* = 0.02; Sec23: *t*(3) = 3.8, *p* = 0.016] (**Figure 4C**). However, in WT cells (**Figure 4C**), when treated with BFA, we see a significant increase in all COPII components compared to control or untreated levels [*f*(3) = 18.2929, *p* = 3.117e^-08^], this overall increase in expression of these genes is not observed in the *Luman* KO cells; [*f*(3) = 1.801, *p* = 0.1797] (**Figure 4C**). This indicates that the *Luman* KO cells exhibit a defective response to the BFA that is normally seen in WT cells.

## Discussion

Variations in stress sensitivity have been linked to many prevalent diseases and the mechanisms behind differences in stress sensitivity are not well understood. The HPA axis is considered the major stress response system and has been shown to play an important role in precipitating the onset of many prevalent mental disorders; however, recently stress sensitivity has been linked to other mechanisms as well (Chrousos, 2009). Variations in HPA axis reactivity have been linked to changes in specific gene expression, which has been proposed to be caused by chronic stress leading to altered DNA methylation patterns (Quax et al., 2013; Weaver et al., 2017). Stress has also been shown to regulate the expression of retrotransposons in the rat hippocampus via an epigenetic mechanism (Hunter et al., 2015), and even accelerated telomere shortening (Epel et al., 2004). Thus, identifying and examining factors that affect the stress response at both the physiological and cellular level are of great importance to allow us to gain a better understanding of stress sensitivity and the mechanisms behind it. We have previously identified the stress-induced transcription factor LUMAN, as one such factor. Here, we examine the molecular mechanism of LUMANs effect on the HPA axis, showing that it alters GR activity through acting as a cofactor of GR, and possibly altering the secretory capacity of cells under stress.

Previously, we have shown that in the absence of LUMAN the expression of GR increases, whereas circulating CORT levels decrease (Penney et al., 2017). In comparison to the blunted stress response observed in *Luman*-deficient mice, patients suffering from depression show the opposite trend of low GR expression and increasing circulating CORT levels (Pariante and Lightman, 2008). Factors such as LUMAN that modulate stress responsiveness could play a role in the onset or progression of depression, by modulating GR expression and/or activity. Here, we present evidence that LUMAN binds to GR through the NR box binding motif and that this interaction results in increased activity of GR, both in the presence and absence of a ligand (DEX) (**Figure 1A**). When the LxxLL motif was mutated (LxxLL KO), activation was still observed without DEX. Under DEX treatment a dramatic increase in activation was observed to be comparable to that seen with N-terminal LUMAN (**Figure 1A**). This suggests that LUMAN acts as a coactivator of GR through binding at the protein level as well as alternative mechanisms. When activated via DEX, LUMAN translocates to the nucleus, and acts as both a transcription factor binding to the GRE in the promoter of the GR gene (**Figure 3A**), and a cofactor, binding to GR (**Figures 3B,C**). When LUMAN is unable to bind directly to DNA (ΔDBD), there is no activation seen without DEX treatment (**Figure 1A**), suggesting that the ligand-independent activation of GR, seen with N-terminal LUMAN, is facilitated through LUMAN’s transcriptional activity. However, the ΔDBD mutant can still enhance GR activity under DEX treatment. Taken together, these data suggest that LUMAN plays a dual role as a transcription factor, binding to promoter regions in DNA, and as a co-factor of GR, altering the GR-mediated stress response. The Co-activator ability of LUMAN likely functions through ligand-mediated binding to GR through the AF-2 domain, as ΔDBD LUMAN was still able to act as a coactivator in the presence of DEX (Lavery and McEwan, 2005).

Altered GR function has been a consistent finding when LUMAN is deficient, but a paradoxical relationship exists; when LUMAN is deficient, GR activity is enhanced, while here evidence suggests that LUMAN acts as a coactivator of GR. It is important to note that GR is essential during embryonic development, and when it is absent there are many defects including lack of lung maturation and death shortly after birth (Reichardt et al., 1998). Therefore, it is possible that when the expression of an essential co-activator of GR is decreased, such as LUMAN, GR levels increase. When specific NCoAs are absent, such as SRC-1, it has been shown that the activity of the NR is altered (Tetel, 2009). An increase in GR expression would lead to enhanced negative feedback in the HPA axis, resulting in decreased circulating CORT levels (Mizoguchi et al., 2003). Previous GR hyperactivity models have corroborated these findings, showing that early in ontology the HPA axis is able to remodel itself to decrease ACTH and cortisol release to allow for normal basal GR reactivity (Murani et al., 2016). The mechanism through which this remodeling occurs has been suggested to happen at the epigenetic level, leading to the need to identify factors that alter gene expression and even chromatin structure to allow a better understanding of the molecular basis of stress sensitivity (Hunter et al., 2015). This model shows that the HPA axis is a malleable network that is able to change in response to chronic alterations in stress sensing through GR. Each of these characteristic changes in hormone signaling are observed in the *Luman*-deficient mice. However, this is not the only mechanism through with LUMAN can exert its effects and alternative mechanisms need to be explored, including the potential role for LUMAN in altering the secretory capacity under stress.

The HPA axis is the major neuroendocrine system that is responsible for the stress response in mammals (Pariante and Lightman, 2008). This system contains many cell types that have a high secretory demand, particularly during stress. These cells, among others found around the body, are considered “professional” secretory cells, and are capable of secreting thousands of proteins per second (Molinari and Sitia, 2005). To meet this demand, these cells contain a highly developed ER, to allow them to increase the level of protein secretion without triggering ER stress (Sano and Reed, 2013). Protein biogenesis in the ER is coupled to a tightly controlled quality check known are the ER-associated degradation (ERAD) pathways; where misfolded proteins are retained in the ER, and eventually degraded by various pathways (Molinari and Sitia, 2005). The activity of the ERAD-related proteins therefore needs to be adapted to variations in the load of the ER with cargo proteins, or misfolded proteins may accumulate and lead to apoptosis (Fulda et al., 2010). The capacity of ERAD also determines the efficiency of protein secretion by keeping the misfolded proteins at a minimum. It has been suggested that LUMAN is involved in the ERAD pathways and disruption of its function may compromise secretion capacity of the cells (Liang et al., 2006). Therefore, inability to rapidly increase secretion and the resulted ER stress may account for the observed blunted stress response in the LUMAN deficient mice.

Many factors function coordinately to selectively increase secretory capacity in response to rapid demand in the event of stress (Turner et al., 1992). However, the underlying mechanism is not well understood. Therefore, identifying new potential factors that could be involved is important. Previous research has shown that CREB3 family proteins are involved in cellular secretion, including CREBL1, CREB3L2 and the *Drosophila* counterpart CREB A. CREB3L1 is essential for bone formation, through activating the secretion of bone matrix proteins, while CREB3L2 is responsible for the secretion of collagens and other extracellular matrix proteins during normal chondrogenesis (Fox et al., 2010; Chan et al., 2011; Fox and Andrew, 2015). These CREB3 proteins are essential in the secretion pathway and their functions differ primarily due to different expression patterns.

We have identified LUMAN, which is highly expressed in neuroendocrine tissues, as a potential factor that can selectively increase the secretory capacity in cells that play an important role in HPA axis function. Although no significant difference was observed in the stress-induced secretion of CRH between *Luman*-deficient and WT mice, (**Supplementary Figure S3**) this does not necessarily eliminate secretion as a possible mechanism through which LUMAN works. Regulation of CRH secretion is complex and occurs through numerous mechanisms, including immune regulation through interleukin-1β, in addition to neuropeptides such as norepinephrine (NE), serotonin (Wei et al., 2002) and brain derived neurotrophic factor (BDNF) (Jeanneteau et al., 2012). In the *Luman*-deficient mice, there was twofold to threefold lower expression of BDNF in the hippocampus when compared to WT mice (**Supplementary Figure S4**), this could contribute to CRH secretion regulation. Previously we have shown that in the *Luman*-deficient mice there appear to be less storage vesicles in the adrenal medulla, which are believed to contain catecholamines (CA). However, no difference was found in the level of circulating CA, indicating other mechanisms may be at play (Penney et al., 2017). Taking this into account, it is possible that many points of control for CRH secretion are responsible for maintaining normal levels of circulating CRH in the absence of LUMAN. Data collected using LUMAN KO and WT MEF cells suggest that LUMAN increases the secretory capacity through enhancing elements of the UPR during times of high secretory demand. Data show that secretion is stunted in the absence of LUMAN when cellular stress is elicited. Furthermore, the key proteins that make up COPII vesicles do not show the normal stress-induced increase in expression in the absence of LUMAN. This suggests that the COPII components may be downstream targets of LUMAN, which are activated in response to cellular stress, to maintain ER homeostasis during high secretion demand which occurs at the onset of the stress response.

In conclusion, LUMAN alters GR activity through binding GR via the LxxLL motif, and it also acts as a transcription factor, binding GREs independently of GR. Additionally, LUMAN alters the secretory capacity of cells when the secretion demand is high, through altering gene expression of the components of COPII vesicles. We therefore postulate that LUMAN plays dual roles in the stress response, regulating secretion at the cellular level, and acting as a cofactor of GR. It is clear that LUMAN plays key roles in the stress response, altering stress sensitivity; this suggests LUMAN as a potential factor that may be involved in the development of stress-related pathologies.

## Author Contributions

JP designed the study, executed the experiments, analyzed and interpreted the data, wrote and edited the manuscript, made the figures, and submitted the paper. TT helped to design Co-IP experiments, completed the Co-IP experiments, and edited the paper. NM and RL helped in designing the study and interpreting data along with edits.

## Conflict of Interest Statement

The authors declare that the research was conducted in the absence of any commercial or financial relationships that could be construed as a potential conflict of interest.

## Supplementary Material

FIGURE S1 Confirmation of expression of ΔAD in HEK293 cells.

FIGURE S2 Low brefeldin A (BFA) concentration induces LUMAN cleavage. Luman knockout and wildtype MEF cells were treated with 200nM BFA for 3 h, after which they were collected and run on an SDS gel and analyzed via Western blot for LUMAN.

FIGURE S3 Circulating CRH levels. Blood was taken through cardiac puncture, spun down, serum removed and frozen until a CRH ELISA was performed. *N* = 8; *P*-value > 0.1 by a two tailed *t*-test.

FIGURE S4 BDNF expression in the hippocampus. Luman-deficient mice have significantly lower BDNF expression when compared to WT mice. Measured via Q-RTPCR from RNA extraction from the hippocampus. ^∗^*p* = 0.003, *T* = 7.08, *DF* = 3; calculated by a one-tailed *t*-test and values are mean ± SEM.

FIGURE S5 Immunofluorescence of GR and CREB3. The localization of GR (red-594) and CREB3 (green-488) in the presence and absence of DEX, as well as looking at the mutant CREB3 constructs. Scale bars: 30 μM.

FIGURE S6 Full blots used in **Figure 2**.

FIGURE S7 Co-Staining of VSVG (488) and GM130 (594) to show Golgi localization for **Figure 3**.

TABLE S1 Primer sequences used. Primer efficiencies were completed for each set of primers and we determined to be between 90–110%.
