# Supplementary figures and images for: LUMAN/CREB3 Plays a Dual Role in Stress Responses as a Cofactor of the Glucocorticoid Receptor and a Regulator of Secretion
Source: Front Mol Neurosci. 2018 Sep 26;11:352. doi: 10.3389/fnmol.2018.00352 (PMC6179040; doi:10.3389/fnmol.2018.00352)

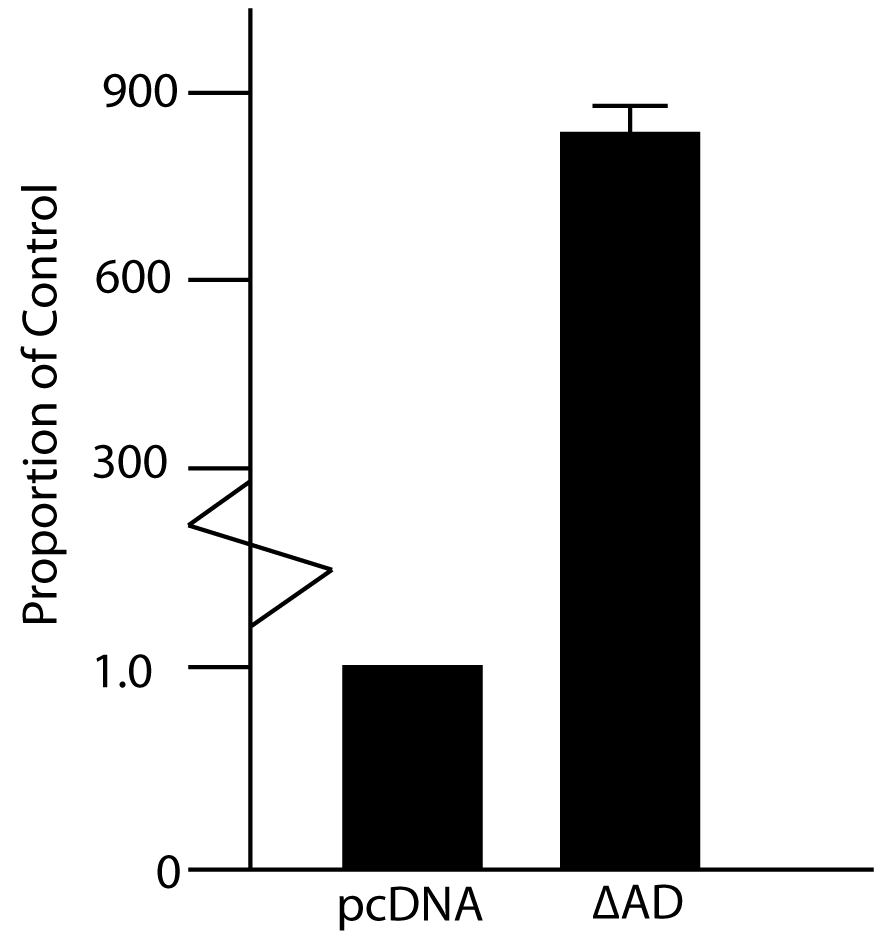

Supplement: FIGURE S1 — Confirmation of expression of ΔAD in HEK293 cells. [file Image_1.TIF]

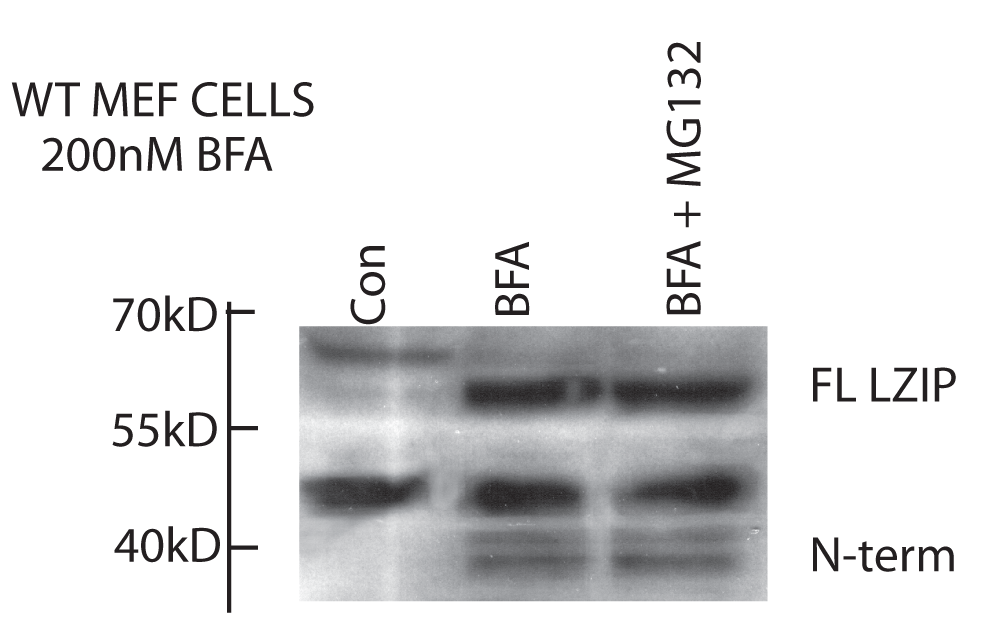

Supplement: FIGURE S2 — Low brefeldin A (BFA) concentration induces LUMAN cleavage. Luman knockout and wildtype MEF cells were treated with 200nM BFA for 3 h, after which they were collected and run on an SDS gel and analyzed via Western blot for LUMAN. [file Image_2.TIF]

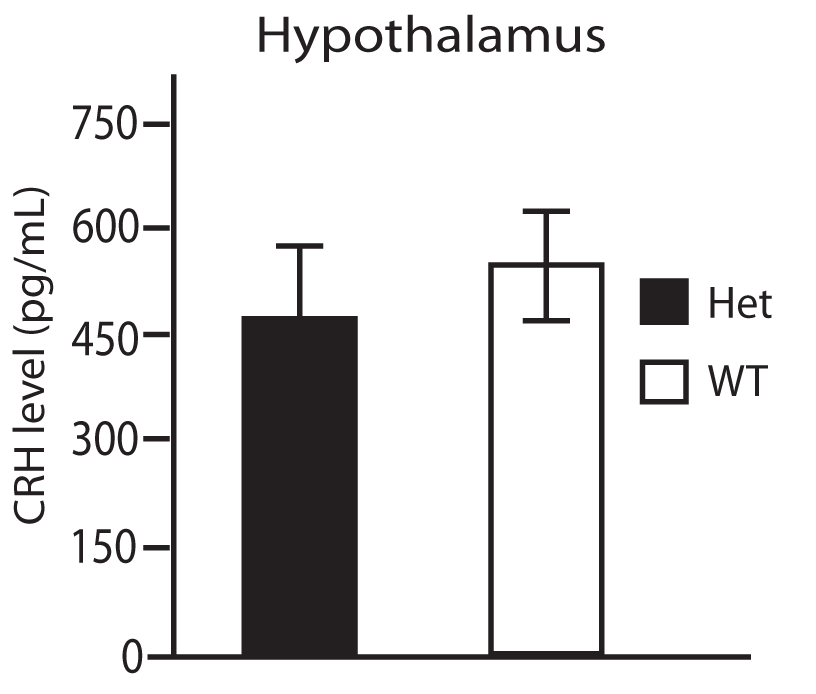

Supplement: FIGURE S3 — Circulating CRH levels. Blood was taken through cardiac puncture, spun down, serum removed and frozen until a CRH ELISA was performed. N = 8; P-value > 0.1 by a two tailed t-test. [file Image_3.TIF]

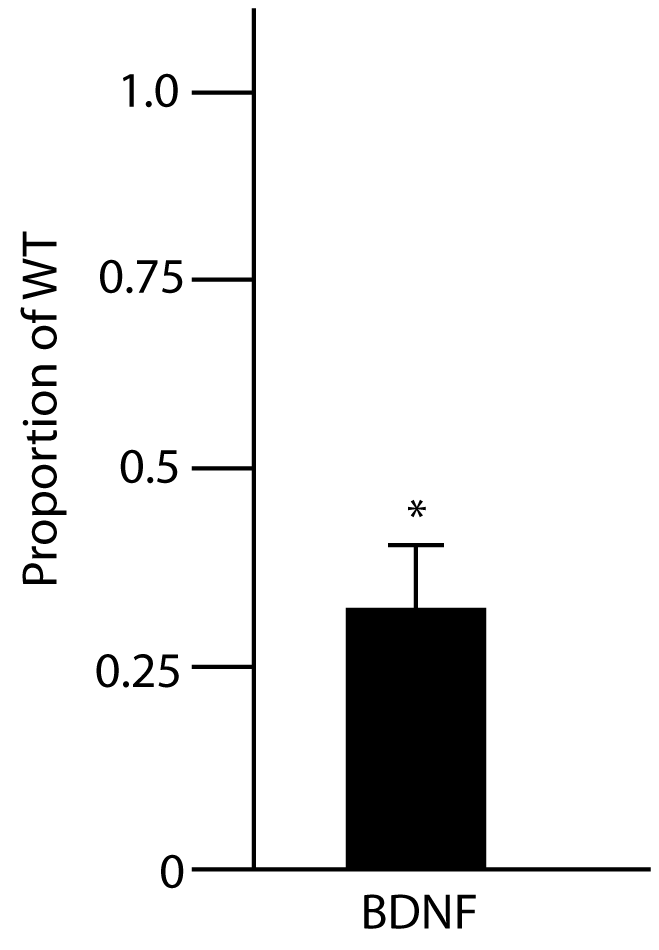

Supplement: FIGURE S4 — BDNF expression in the hippocampus. Luman-deficient mice have significantly lower BDNF expression when compared to WT mice. Measured via Q-RTPCR from RNA extraction from the hippocampus. ∗p = 0.003, T = 7.08, DF = 3; calculated by a one-tailed t-test and values are mean ± SEM. [file Image_4.TIF]

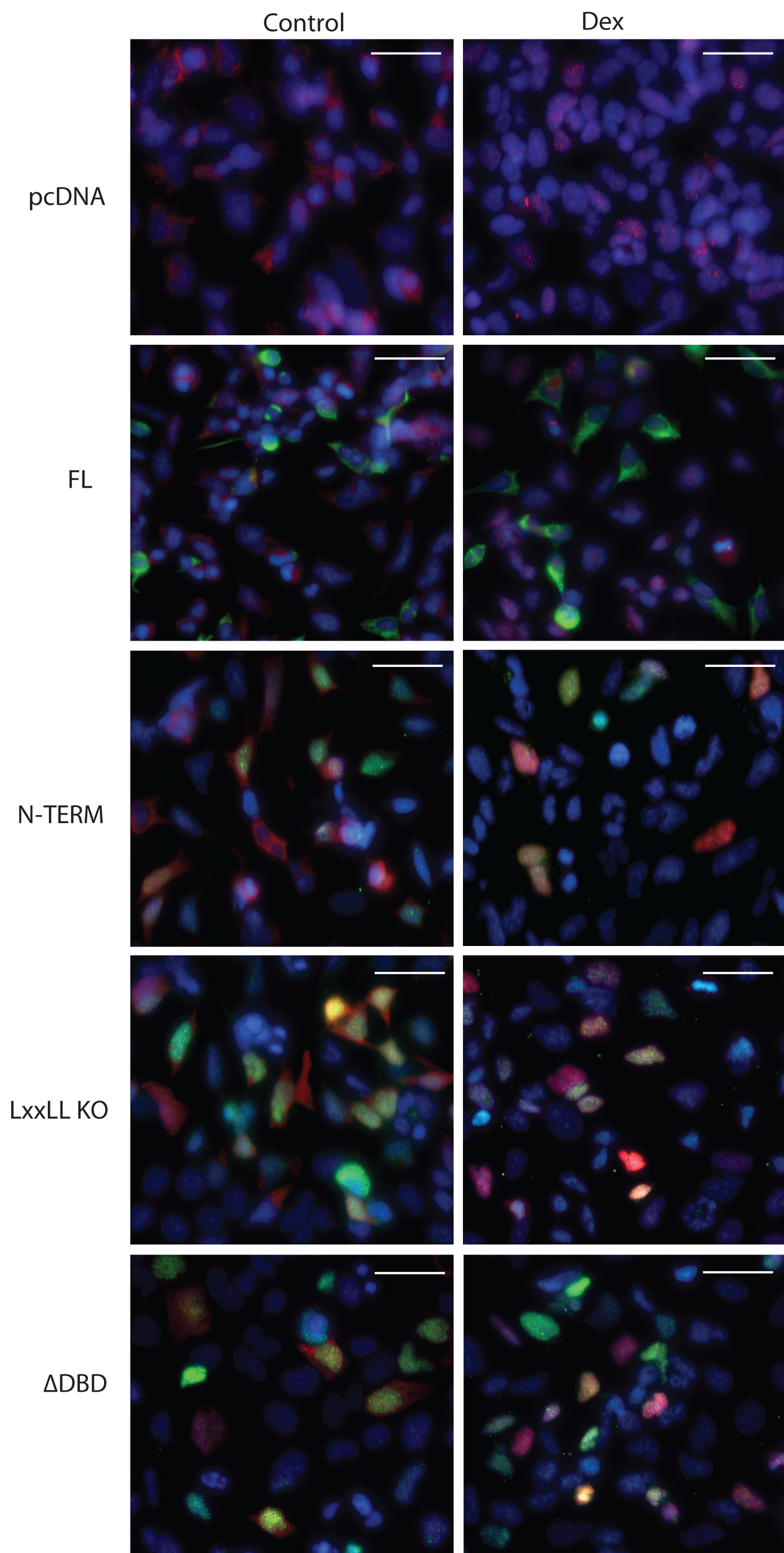

Supplement: FIGURE S5 — Immunofluorescence of GR and CREB3. The localization of GR (red-594) and CREB3 (green-488) in the presence and absence of DEX, as well as looking at the mutant CREB3 constructs. Scale bars: 30 μM. [file Image_5.TIF]

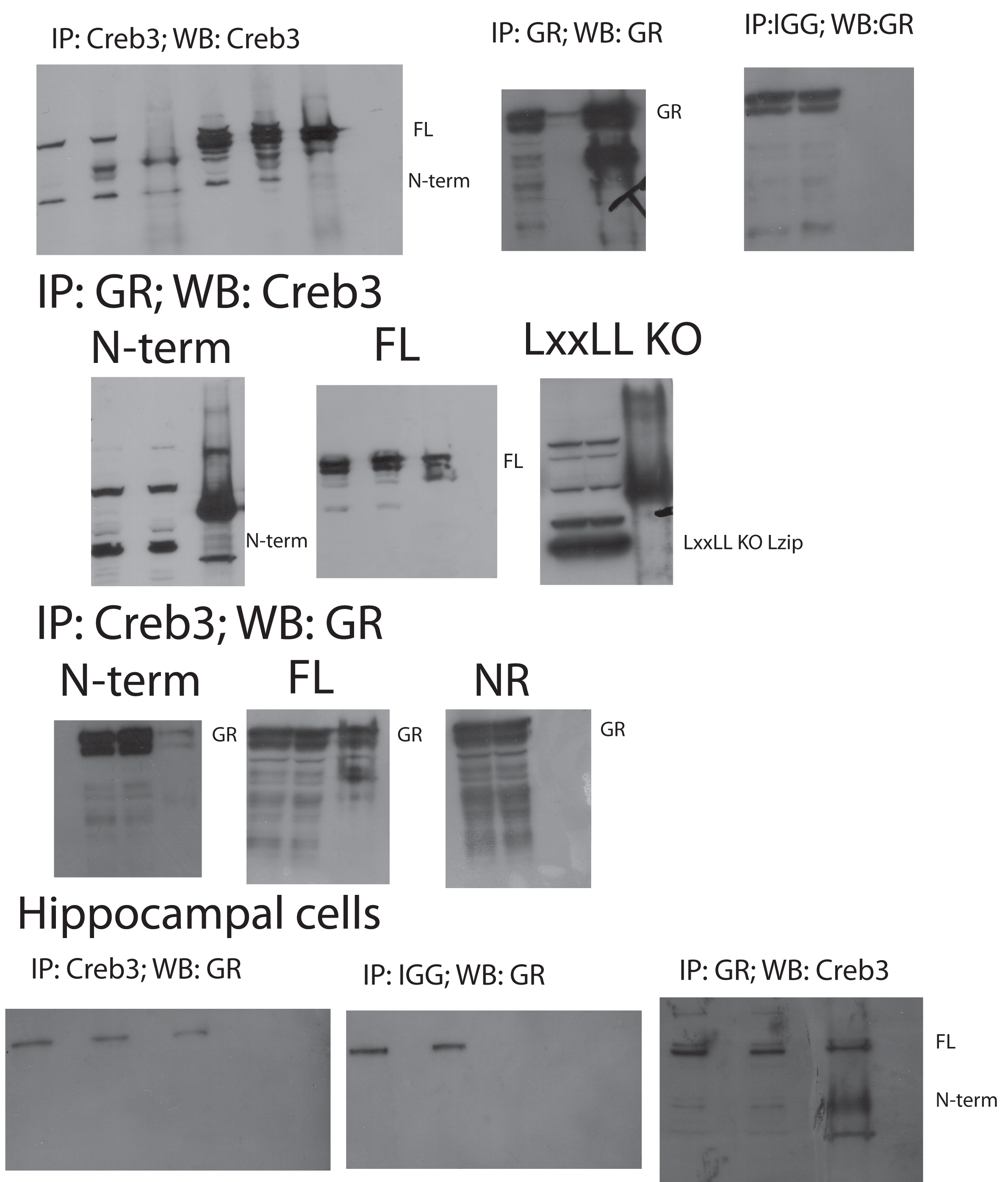

Supplement: FIGURE S6 — Full blots used in Figure 2. [file Image_6.TIF]

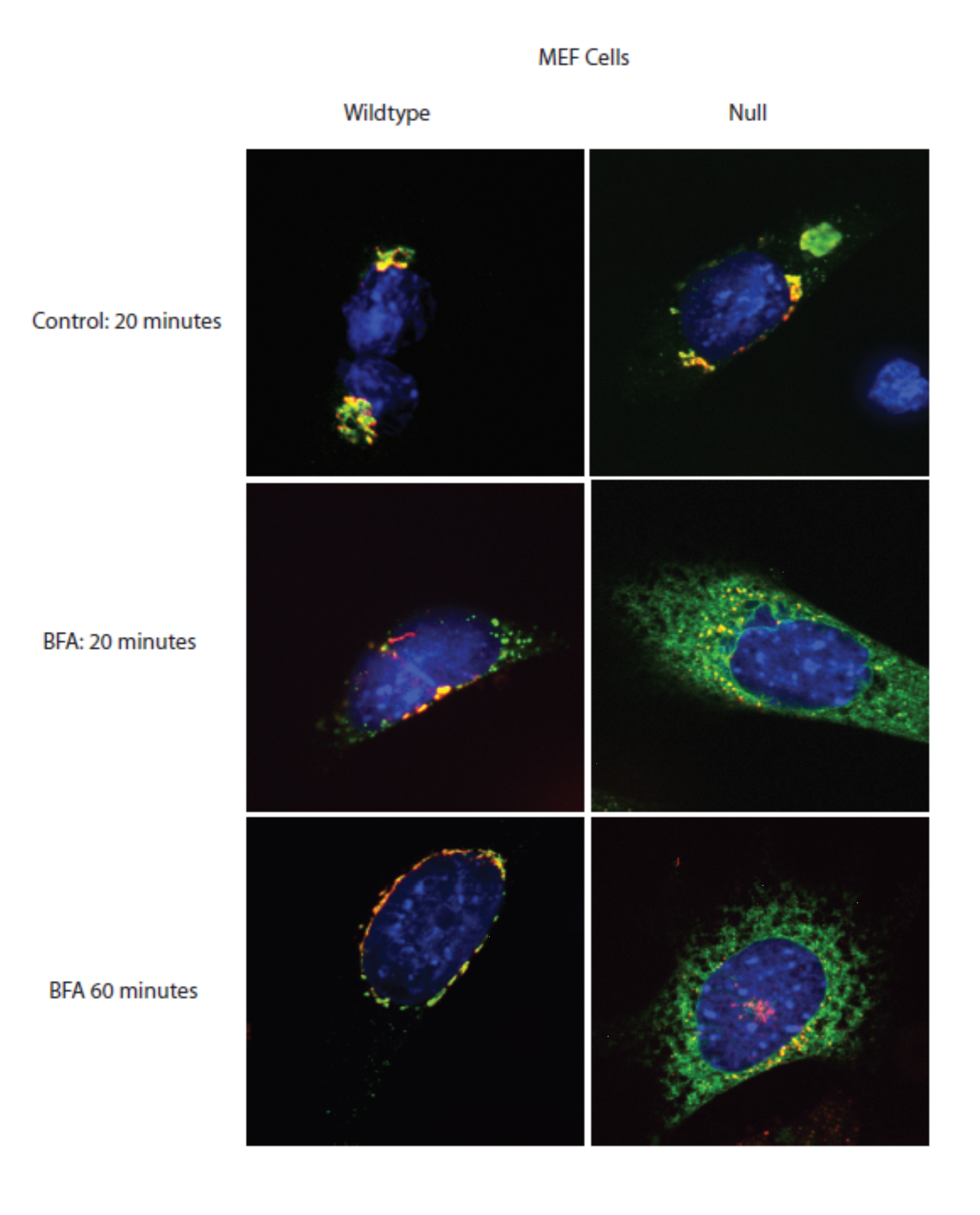

Supplement: FIGURE S7 — Co-Staining of VSVG (488) and GM130 (594) to show Golgi localization for Figure 3. [file Image_7.TIF]
