# Supplementary material for: LUMAN/CREB3 Plays a Dual Role in Stress Responses as a Cofactor of the Glucocorticoid Receptor and a Regulator of Secretion
Source: Front Mol Neurosci. 2018 Sep 26;11:352. doi: 10.3389/fnmol.2018.00352 (PMC6179040; doi:10.3389/fnmol.2018.00352)
Supplement: TABLE S1 — Primer sequences used. Primer efficiencies were completed for each set of primers and we determined to be between 90–110%. [file Table_1.docx]

| **Gene** | **Forward (5’-3’)** | **Reverse (5’-3’)** |
| --- | --- | --- |
| GR | CCACTGCAGGAGTCTCACAA | AGCAGTGACACCAGGGTAGG |
| FKBP5 | TCCAAGTCCTTTCGCAGACT | TACCCACTCTCCACACCACA |
| SGK1 | TCTGCAATGTGCCTTTTCTG | ATAGCACACTCACGCCACTG |
| Β-Tubulin | CTGGAGAAGCCAGGTTGACA | TTTCTCTGGACCACCAACGC |
| 18s rRNA | GTAACCCGTTGAACCCCATT | CCATCCAATCGGTAGTAGCG |
| GAPDH | TACCCCCAATGTGTCCGTCG | CCTGCTTCACCACCTTCTTG |
| Sec31 | CTGGAGCATCCTTCCAGCAT | CAGGCGGTGTACCTGTTGTT |
| Sec13 | TCTGGAAGGAGGAAAACGGC | GCCAGGATCAGGCCATAGTC |
| SAR1 | CTCGGGCCGTTGTAAGCATC | GTAGAGTCCTAGGAACTGGAGCA |
| Sec 23 | GTCACGGAGCTTCTAGACAGAAAT | TTCCAACTGAACCGGACCC |
| Sec 24 | TCCGATGATGAGGAGGAGCA | TCGTAACTGTTGGGCAAGGG |
| SIRT1 | GAGTCCATCGGTCAACAGGA | ACCATACAGGCCAAACGGG |
| SLC2A1 | CAAGCAAGCATAGAAGCCCAG | TCCAACACGAACCTCTGAACC |
| FOXO1 | AGTGGATGGTGAAGAGCGTG | GAAGGGACAGATTGTGGCGA |
| ACSS2 | CTGCTTCCAGTCAGACCCTC | GAGGTCTCCAAGCCCAGAAC |
| SQLE | AAGCTTGGTCCTCAAGTCCAC | GAGTCCACGTGCAGTCAAGT |
| CYP3A4 | CAAGGCACCTCCCACGTATG | ATCACTGTGACCCTTGGGGA |
|  |  |  |
| **ChIP Primers** | | |
| Per1 | GGGTTGGGGGAGGCGCCAA | GGCGGCCAGCGCACTAGG |
| Sec 23 (UPRE) | GATCCCTGCGATAGTCGTGG | GAGGACAAGACACGAGACGG |
| FKBP5 (GRE) | AGTACCCAACAGAGGTCAGA | TGTGGATACAAAATATTCCA |
| DDC | GCCCTGGGGAATGACATCAGC | AGCTCAGCCAAGCAAGTCGAAG |
| GAPDH (PROM) | ACTGAGCAAGAGAGGCCCTA | TATGGGGGTCTGGGATGGAA |
| Sec13 (UPRE) | GATCCCTGCGATAGTCGTGG | GAGGACAAGACACGAGACGG |

**Sup. Table. 1.** Primer sequences used. Primer efficiencies were completed for each set of primers and we determined to be between 90-110%.
